# Exclusive Enteral Nutrition versus Infliximab in Inducing Therapy of Pediatric Crohn's Disease

**DOI:** 10.1155/2017/6595048

**Published:** 2017-08-08

**Authors:** Youyou Luo, Jindan Yu, Jingan Lou, Youhong Fang, Jie Chen

**Affiliations:** Department of Gastroenterology, The Children's Hospital, School of Medicine, Zhejiang University, Hangzhou, Zhejiang Province 310051, China

## Abstract

**Aim:**

To compare the effectiveness of exclusive enteral nutrition (EEN) and infliximab (IFX) therapy in pediatric Crohn's disease (CD).

**Methods:**

In a prospective study of children initiating EEN or infliximab therapy for CD, we compared clinical outcomes using the pediatric Crohn's disease activity index (PCDAI), growth improvement, endoscopic mucosal healing, and adverse effects. Data were measured at baseline and after 8 weeks of therapy.

**Results:**

We enrolled 26 children with CD; of whom, 13 were treated with infliximab, 13 with EEN. Clinical response (PCDAI) reduction ≥ 15 or final PCDAI ≤ 10 was achieved by 83.3% in the EEN group and 90.9% in the IFX group. Body mass index for age (BMIFA) *z*-scores were significantly increased in both groups (*P* < 0.05). No significant differences were observed in PCDAI, height for age (HFA), or BMI recovery between two groups. Adverse effects were detected in 30.7% on infliximab and 0% on EEN. Mucosal healing was achieved in 71.4% cases in the EEN group versus 85.7% in the IFX group.

**Conclusion:**

EEN provided similar improvements as IFX in clinical symptoms, mucosal healing, and BMI. EEN therapy has less adverse effects when compared with IFX. This trial is registered with the Clinical Registration Number: ChiCTR-OON-17010834.

## 1. Introduction

The treatment strategies for Crohn's disease (CD) are complex. Most of the medications have multiple side effects. Traditionally, the aims of therapy have been to relieve symptoms, optimize growth, and improve quality of life while minimizing drug toxicity [1]. Recently, mucosal healing (MH) has emerged as a major therapeutic goal in clinical trials in inflammatory bowel disease (IBD). Accumulating evidence revealed that MH may change the natural course of the disease by maintaining clinical remission, reducing the need for surgery, and increasing the steroid-free rate [2]. Therefore, symptoms, growth, and MH are role issues to evaluate the efficacy of the treatment.

Infliximab, a monoclonal antibody-targeting tumor necrosis factor (TNF), is one of the primary treatment strategies for active pediatric CD, while exclusive enteral nutrition (EEN) therapy is suggested as first-line therapy to induce remission [1]. Both infliximab and EEN have shown advantages in inducing remission and improving growth and MH [3–6]. However, acute infusion reactions (AIR), infections, and risk of malignancy are the main concern of patients who are receiving infliximab as induction remission therapy. Meanwhile, patients' poor compliance may lead to EEN treatment failure. Thus, the balance between efficacy, risk of side effects, and patients' compliance is an important consideration in choosing therapeutic regimens.

Since rare studies showed comparative effectiveness of those approaches, we prospectively compared the efficacies, growth improvements, and adverse effects of the two regimens in children with newly diagnosed CD.

## 2. Patients and Methods

Children and adolescents less than 18 years of age were enrolled at the time of initiation of EEN or infliximab for treatment of active CD at the Children's Hospital, School of Medicine, Zhejiang University, Hangzhou, China. This hospital is one of the major referral centers for children with IBD in Eastern China. To be included, patients in the study must be newly diagnosed and be followed up by the IBD clinic for at least 2 months. The exclusion criteria includes the following: (1) the pediatric Crohn's disease activity index (PCDAI) < 10; (2) treatment with anti-TNF therapy within 8 weeks of starting EEN; (3) treatment with EEN therapy within 8 weeks of starting infliximab; (4) intestinal surgery before initiation of EEN or infliximab; (5) treatment with probiotics within 2 weeks of initiating EEN; (6) previous administration of corticosteroids and immunosuppressive drugs before CD diagnosis. Occurrence of adverse events and patients' compliance were recorded. The study protocol was approved by the Ethics Committee of Zhejiang University, in Hangzhou, China. All pediatric informed consents were provided by participants' parents/guardians.

Data were collected at baseline and week 8, including basic demographics, history, physical examination, the PCDAI, and laboratory findings. The erythrocyte sedimentation rate (ESR) and hemoglobin and serum albumin levels were collected in laboratory results. The disease phenotypes were classified by the Paris classification [7]. The findings of ileocolonoscopy were evaluated by endoscopists according to Crohn's disease endoscopic index of severity (CDEIS) [8]. Clinical remission was defined as PCDAI ≤ 10 points. Clinical response was defined as a reduction in PCDAI by ≥15 points or final PCDAI ≤ 10. Endoscopic complete remission was defined as a CDEIS score ≤ 3 points. A decrease in CDEIS score of >5 points meant response in endoscopic appearance [9]. The choice of EEN or infliximab was based on the patients' and physicians' preference. Patient compliance and side effects of both regimes were recorded.

## 3. Statistical Analysis

Statistical analysis was performed using SPSS 17.0 (SPSS Inc., USA). Data are presented as mean ± medians (interquartile range) according to distribution normality. Parametric values were compared by use of the *t*-test method. The chi-squared test was used for categorical variables. Comparisons were made using 2-sided significance levels of *P* < 0.05.

### 3.1. Results

### 3.2. Study Population at Baseline

Twenty-six participants were enrolled in the study, with 13 initiating EEN and 13 infliximab (Table 1). Age and gender distribution were similar between the 2 groups. Disease duration, ESR, hemoglobin, serum albumin, PCDAI, and CDEIS were similar between the 2 groups. Disease durations of 3 children in the IFX group and 2 children in the EEN group are longer than 12 months. In the IFX group, three cases received incision of perianal abscess. One patient did appendicectomy before CD. All the patients did not receive EEN, biologics, immunosuppressive therapy, or mesalamine before the initial diagnosis was made. Ileocolonic disease and colonic and small intestinal diseases were the commonly seen disease locations in the IFX group. While in the EEN group, only ileocolonic disease (46.2%) was the most frequently seen disease location. No colonic disease was detected in the EEN group. No structuring and penetrating disease was found in both 2 groups except 1 case in the EEN group, which is structuring disease, defined as B2 in Paris classification. Four subjects in the IFX group were found to have perianal diseases (3 with perianal fistulas and 1 with perianal abscess). The EEN group saw no perianal diseases.

### 3.3. Disease Therapy in the 2 Groups

All of the 13 subjects in the EEN group were on Nutren Junior (Nestle), which is a polymeric formula. The average daily caloric intake was 110.0 ± 9.1 kcal per kilogram. EEN was given orally in all patients. One subject discontinued formula feeding because of poor tolerance of feeds. Patients on IFX were treated with a three-dose induction scheme at 0, 2, and 6 weeks. Each dose was 5 mg/kg. 13 subjects completed the induction scheme except for two. One stopped the third dose due to disease flare and sepsis. One discontinued IFX infusion due to seizure after the third dose was initiated.

### 3.4. Clinical Evaluation

After 8 weeks, the remission rate was 83.3% and 90.9% in the EEN and IFX groups, respectively. Ninety-two percent of patients in the EEN group and 91% of those in the IFX group had response to each treatment. The PCDAI scores in both groups were significantly decreased when they were compared with those in the baseline (*P* < 0.001). No significant difference of the change in PCDAI was detected between the two groups. The average values of ESR, hemoglobin, and serum albumin in the EEN and IFX groups are 16 mm/H, 129 g/L, and 43.7 g/L and 23.5 mm/H, 120.2 g/L, and 42.5 g/L, respectively. No significant differences of ESR, hemoglobin, and albumin levels between the two groups were found at the endpoint in the study.

### 3.5. Mucosal Healing

Fourteen patients repeated their colonoscopy after 8 weeks. Among those, 7 individuals were in the EEN group. At the endpoint of the study, 5 cases in the EEN group versus 6 in the IFX group achieved endoscopic complete remission, while 3 versus 2 characters had their CDEIS less than 1. All of the 14 cases had endoscopic response except for two in the EEN group, who had their CDEIS increased.

### 3.6. Growth Evaluation

No significant differences were found between the 2 groups on baseline height for age (HFA) *z*-score and body mass index for age (BMIFA) *z*-score. After 8 weeks of treatment, both of the 2 groups saw a remarkable increase in the BMIFA *z*-score (Table 2). However, the changes of the BMIFA *z*-score, as well as that of the HFA *z*-score, were similar in the 2 groups. When compared with the baseline, the HFA 𝑧-score did not change significantly in both groups.

### 3.7. Adverse Effects

No adverse effects were observed in the EEN group. However, four cases (30.7%) in the IFX group had side effects. Among those, two patients had infusion reaction, including dyspnea, vomiting, coughing, and cyanosis. Symptoms were relieved after the doctors stopped IFX infusion and administered methylprednisolone. One patient had seizure when she was on the third-dose infusion. One patient had recurrent upper respiratory infection.

## 4. Discussion

Despite good efficacy and patients' compliance, IFX usually meets the concern of increasing the rates of malignancies and infections from the parents. For some parents, they are more willing to accept treatment strategies with less side effects. However, those are based on the effectiveness of the treatment strategy and patients' adherence. In recent years, numerous studies showed good results in inducing clinical and endoscopic remission of luminal CD [6, 10–12]. Most of the studies compared the efficacy of different formulas and EEN, PEN versus steroids. The comparison between EEN and IFX therapy on CD was rarely reported [13]. Herein, we present the comparative clinical/endoscopic improvement and nutritional change between EEN and IFX therapy. This study showed that EEN achieved similar efficacy, less side effects, but lower patients' compliance, when compared with IFX.

EEN was known to have up to 90% clinical remission rate in inducing remission therapy on newly diagnosed pediatric luminal CD [6, 10, 14]. It is well established that EEN can relieve clinical symptoms and normalize laboratory parameters associated with active CD in children. In two meta-analysis studies, EEN was demonstrated as having the same effectiveness as corticosteroids in inducing disease remission in pediatric CD [15, 16]. With respect to corticosteroids, EEN has its advantage on mucosal healing, restoration of nutritional status, bone health, and liner growth in children [1]. In view of those benefits from EEN, Lee et al. compared the clinical effectiveness of EEN, partial enteral nutrition (PEN), and anti-TNF therapy. The results revealed that the remission rate between EEN and TNF has no significant difference (88% and 84%, resp.) [13]. Our study demonstrated similar outcomes.

Mucosal healing, associated with long-term sustained remission, fewer complications, and surgeries, is one of the treatment goals in pediatric CD. Pediatric data concerning mucosal healing were limited. In a recent retrospective study including 66 children with moderate-to-severe CD on IFX, 22.7% of those reached mucosal healing [4]. EEN was reported to induce early mucosal remission in up to 33% CD children in previous studies [10, 17]. In this study, ten children repeated their colonoscopy after 8 weeks of treatment. Four out of 5 children in each group showed endoscopic complete remission, indicating similar results in the mucosal healing rate of IFX and EEN therapy. Since we have different standard to evaluate mucosal healing, it is difficult to compare those results.

With respect to adult cohort, pediatric CD has specific features. Failure to thrive, delayed puberty, low bone mineral density, and weight loss frequently affect CD patients. The pathogenesis of these symptoms are complex, including reduced dietary intake, increased gastrointestinal nutrient losses, and increased energy requests due to active disease and treatment of corticosteroids [18]. All these reasons demonstrate the importance of nutritional therapy in pediatric CD. Since EEN was first used as inducing remission therapy in pediatric CD, it has been reported that EEN can help the patients to gain weight and height [6, 11]. Meanwhile, IFX has similar abilities to increase weight, but opinions were different about its efficacy of catch-up growth [19]. However, to our knowledge, no study compared their treatment efficacy on weight and height gain in pediatric CD cohort. Our study demonstrated that EEN and IFX had similar effectiveness to improve BMI in CD children. But, they might not improve height in a short term. Since the sample in the study was small and the subjects were followed up only for 8 weeks, a larger sample with long-term follow-up is needed to certify the outcomes.

There are several limitations in this study. Since the incidence of pediatric Crohn's disease is lower than western countries, the sample we collected in the study is small. It would be ideal if a larger number of multicenter data were enrolled. Secondly, this is a nonrandomized study. There was a selection bias when clinicians and parents chose treatment regimen at the beginning. Therefore, larger randomized, controlled sample studies should be encouraged.

## 5. Conclusions

Our study revealed that EEN might have similar abilities with IFX to induce remission of pediatric luminal CD. Both EEN and IFX can improve body weight, but not height, in a short term. To our knowledge, this is the first study comparing the efficacy of nutritional status between EEN and IFX.

## Figures and Tables

**Table 1:** Demographic and clinical data at baseline of the treatment groups.

	EEN	IFX
*N*	13	13
Sex (M, %)	9, 69.2%	6, 46.2%
Age, years (range)	11.9 (5.4–15.3)	11.7 (1.1–13.7)
PCDAI (mean ± S.D.)	26.0 ± 9.3	29.5 ± 11.7
Disease duration (months, range)	12.6 (1.1–91.7)	12 (1.0–100.1)
Disease location (%)		
Ileal (L1)	23.1	15.4
Colonic (L2)	0	38.5
Ileocolonic (L3)	46.2	38.5
L4a	7.7	0
L4b	38.5	23.1
L4a + L4b	30.8	38.5
Disease behavior (%)		
B1	92.3	100.0
B2	7.7	0
Perianal diseases (%)	0	30.8
ESR (mm/H)	38.0 ± 26.3	40.5 ± 33.0
HB (g/L)	113.8 ± 12.3	109.8 ± 11.9
Albumin (g/L)	37.3 ± 4.8	33.5 ± 7.3

EEN: exclusive enteral nutrition; IFX: infliximab; PCDAI: pediatric Crohn's disease activity index; ESR: erythrocyte sedimentation rate; HB: hemoglobin.

**Table 2:** Comparison of growth recovery in EEN and IFX groups.

	EEN (*n* = 12)	IFX (*n* = 11)
HFA *z*-score		
Baseline	−0.7 ± 1.6	−0.1 ± 1.2
After 8 weeks	−0.5 ± 1.6^#^	−0.2 ± 1.1
Change in HFA *z*-score	0.1 ± 0.4	−0.1 ± 0.3
BMIFA *z*-score		
Baseline	−1.5 ± 1.3	−1.9 ± 1.1
After 8 weeks	−0.4 ± 1.3^∗^	−0.7 ± 0.9
Change in BMIFA z-score	1.2 ± 0.7	1.2 ± 0.8

EEN: exclusive enteral nutrition; IFX: infliximab; PCDAI: pediatric Crohn's disease activity index; HFA: height for age; BMFAI: body mass index for age. ^#^*P* < 0.05; ^∗^*P* < 0.01.
